# Graph models of brain state in deep anesthesia reveal sink state dynamics of reduced spatiotemporal complexity

**DOI:** 10.1162/NETN.a.27

**Published:** 2025-10-30

**Authors:** James Barnard Wilsenach, Charlotte M. Deane, Gesine Reinert, Katie Warnaby

**Affiliations:** Alan Turing Institute, London, UK; Department of Statistics, University of Oxford, Oxford, UK; Oxford Centre for Functional MRI of the Brain, University of Oxford, Oxford, UK

**Keywords:** Dynamic brain state models, Consciousness, Complexity, Spatiotemporal community detection, Hidden Markov models, Anesthesia

## Abstract

Anesthetisia is an important surgical and explorative tool in the study of consciousness. Much work has been done to connect the deeply anesthetized condition with decreased complexity. However, anesthesia-induced unconsciousness is also a dynamic condition in which functional activity and complexity may fluctuate, being perturbed by internal or external (e.g., noxious) stimuli. We use fMRI data from a cohort undergoing deep propofol anesthesia to investigate resting state dynamics using dynamic brain state models and spatiotemporal network analysis. We focus our analysis on group-level dynamics of brain state temporal complexity, functional activity, connectivity, and spatiotemporal modularization in deep anesthesia and wakefulness. We find that in contrast to dynamics in the wakeful condition, anesthesia dynamics are dominated by a handful of sink states that act as low-complexity attractors to which subjects repeatedly return. On a subject level, our analysis provides tentative evidence that these low-complexity attractor states appear to depend on subject-specific age and anesthesia susceptibility factors. Finally, our spatiotemporal analysis, including a novel spatiotemporal clustering of graphs representing hidden Markov models, suggests that dynamic functional organization in anesthesia can be characterized by mostly unchanging, isolated regional subnetworks that share some similarities with the brain’s underlying structural connectivity, as determined from normative tractography data.

## Author Summary

The imperturbability of subjects undergoing general anesthesia is surgically important and is well studied in the neuroscience of consciousness. However, research is ongoing on how this perturbation resistance is realized dynamically, on the level of changes in functional connectivity. Here, we demonstrate a possible explanation in terms of brain states of reduced functional complexity to which anesthetized subjects repeatedly return. These states act as sinks that reduce the overall temporal complexity of anesthesia dynamics in a manner that may depend on surgically relevant susceptibility and age-related factors. Using a novel graph-based spatiotemporal clustering method, we show that anesthesia induces not just temporal but also spatiotemporal reductions in complexity, resulting in states that are functionally fragmented into unchanging, anatomically restricted subregions with minimal information sharing between these fragments.

## INTRODUCTION

General anesthesia is an invaluable surgical tool that reduces the overall risk of complications and shields patients from immediate pain while being generally safe and effective (Urban & Bleckwenn, 2002). However, anesthetic susceptibility and risks are patient specific and may include impaired respiration and heart function (Nunn, 1990), delayed recovery of consciousness, and postoperative delirium in overanesthesia (Bryson & Wyand, 2006; Fritz et al., 2016). Overanesthetization has been associated with cognitive decline in older patients who are also at higher risk of postoperative delirium (Sevenius Nilsen, Juel, & Marshall, 2019; Wu, Zhao, Weng, & Ma, 2019). Conversely, underanesthetization may risk psychological trauma through postsurgical recollection of pain and helplessness, especially in combination with commonly administered muscle relaxants (Aceto et al., 2013; Schwender et al., 1998; Urban & Bleckwenn, 2002). These factors motivate the importance of understanding how the brain dynamically maintains unconsciousness under sustained anesthesia when subjected to passive or, in the case of surgery, powerful forms of external stimulation (Bonhomme et al., 2019).

More generally, understanding dynamic functional changes in neural configuration, or *brain state*, has been key to describing the processes involved in the transitions between and maintenance of different conscious and unconsciousness conditions (He, Das, Hotan, & Purdon, 2023; Mediano et al., 2023; C. Zhang et al., 2024). A popular method for determination of brain state space models has been hidden Markov models (HMMs), with applications to sleep (Stevner et al., 2019; Yang et al., 2024), to disorders of consciousness (DOCs) (Bai et al., 2021), and to anesthesia (Liu et al., 2022). In HMMs, multiple signals of brain activity are generally modeled as arising from discrete hidden states; see Supporting Information Section S1 for a brief introduction. These states provide a snapshot of functional activity (FA) and connectivity that subjects transition between, probabilistically and dynamically, to support the maintenance of a condition such as anesthesia-induced unconsciousness or resting wakefulness (Vidaurre et al., 2016). HMMs are appealing in part because of this rich spatiotemporal representations of dynamics that depend on underlying persistent and stable conditions such as in a resting state (Leroux, 1992). Stability dependence differentiates HMMs from more sophisticated models of brain state that may be applied to nonstationary behaviors, usually at the cost of the richness of temporal dynamics in the model (Ito, Yang, Laurent, Schultz, & Cole, 2022; Parks et al., 2024; Rukat, Baker, Quinn, & Woolrich, 2016). We focus our study on HMM analysis of resting brain state dynamics in both wakefulness and anesthesia-induced unconsciousness in order to better understand the dynamic properties that support its stability and maintenance.

HMMs and other state space models have been used to demonstrate a general decrease in temporal (Demertzi et al., 2019; Luppi et al., 2024; Stevner et al., 2019) and signal-based complexity measures (Liang et al., 2024; Ruffini, 2017) during unconsciousness, including under most anesthetics, with the possible exception of ketamine (Li & Mashour, 2019). Of these measures, Kolmogorov complexity is thought to be a possible neural correlate of consciousness and is notable for its strong links to the Integrated Information and Global Workspace theories (Fan, Yeh, Chen, & Shieh, 2011; Marshall, Gomez-Ramirez, & Tononi, 2016; Ruffini, 2017), although its accurate quantification in practice in particular in recordings of brain activity remains computationally and mathematically intractable (Sevenius Nilsen et al., 2019). Using state space models, however, the Kolmogorov complexity can be approximated by the *entropy* or *information rate* (Galatolo, Hoyrup, & Rojas, 2010). This approximation has been used to demonstrate a general decrease in the dynamic reachability or realizability of states during various forms of unconsciousness when compared with wakefulness (Barttfeld et al., 2015; Castro et al., 2024; Demertzi et al., 2019; Li, Vlisides, & Mashour, 2022; Mediano et al., 2023). We corroborate these findings by training HMMs that are specific to each condition, wakefulness and deep anesthesia, and propose that certain condition-specific states may act as low-complexity attractors or *sinks*, which are disproportionately responsible for this reduction in complexity.

Brain states can be considered as graphs connecting brain regions, with edges depending on state-specific functional connectivity connectivity between these regions (Vidaurre et al., 2016). This representation has helped to establish the minimal resting-state network features, which persist to support unconsciousness dynamics (Barttfeld et al., 2015; Heine et al., 2012; Luppi et al., 2019). However, consideration of states in isolation fails to incorporate the temporal dependencies in the activity of these subnetworks. Therefore, in analogy to the static spatially defined resting-state networks (Heine et al., 2012), here, we provide a spatiotemporal framework for brain state analysis to understand the spatiotemporal functional modularization of activity in both conditions, wakefulness and anesthesia. To this purpose, we represent a fitted HMM on brain states as a graph, with brain states as nodes, and the transition probabilities (TPs) between brain states taken as edge weights between nodes; the resulting graph on graphs is a hidden Markov graph model (HMGM) as in Wilsenach, Warnaby, Deane, and Reinert (2022). This particular graph on graphs structure allows us to develop new methods to detect modular spatiotemporal subnetworks or communities, incorporating both functional and temporal information. In particular, we detect spatiotemporal functional stratification that occurs in anesthesia-induced unconsciousness but not wakefulness. We also use these methods to corroborate and expand upon previous work on the close links between structure and unconscious brain state connectivity (Barttfeld et al., 2015; López-González et al., 2021; Luppi et al., 2023).

Our results suggest new avenues for exploration in the study of anesthesia, in particular how age and anesthesia susceptibility may modulate patient trajectories both into and out of anesthesia-induced unconsciousness. Moreover, we demonstrate that unconsciousness dynamics may involve effective restrictions on the space of possible brain states through dominant *sink* states. We find indications of the dissolution of important functional modules and the loss of the brain’s capacity for integrating multiple streams of information under propofol-induced deep anesthesia.

## MATERIALS AND METHODS

**Figure 1.**
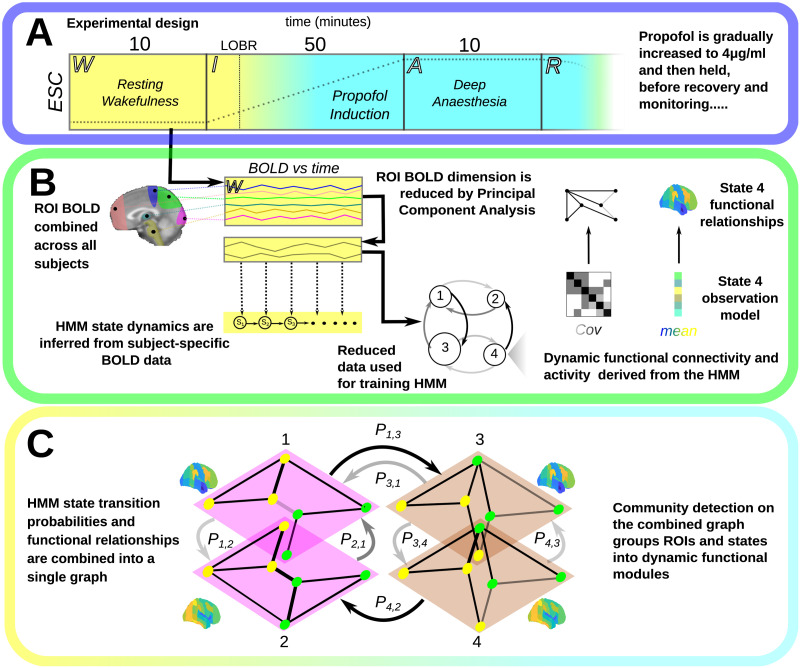
This figure shows our experimental and analysis framework. (A) Subject fMRI is recorded at resting wakefulness (yellow, W); followed by ultraslow propofol induction (I), where the estimated ESC is gradually increased; and loss of behavioral responsiveness during induction is marked with a dotted line, before being held at 4 *μ*g/mL during the deep anesthesia (cyan, Phase A). Anesthesia dosage is then tapered and subjects are allowed to recover, R. (B) ROI BOLD time series across all subjects were first combined and then dimensionality reduced by principal component analysis (PCA). In the framework, brain dynamics arise from a sequence of hidden brain states. Occurrence of the next state is probabilistically dependent on the current state. These probabilities are shown with an arrow (darker, higher probability). Each state encodes expected functional connectivity and activity relationships that are mapped into parcellation space through the state mean (blue, low) and covariance (dark, high) parameters. The analysis is performed again for anesthesia. (D) We combine functional and dynamic information to produce the combined HMGM graph (dark arrows, high probability transition), followed by spatiotemporal community detection.

Figure 1 shows an overview of the experimental setup (Panel A) and the processing of the blood oxygen level-dependent (BOLD) signals, followed by training of the HMM (Panel B); Panel C shows the construction of the HMGM from the trained HMM, and how the brain regions of interest (ROIs) and states can be decomposed in terms of both their functional module or community memberships. This framework builds upon previous work first introduced in Wilsenach (2022) and Wilsenach et al. (2022).

### Data Acquisition and fMRI Preprocessing

BOLD fMRI data were recorded over the course of a four-phase experiment in which 16 initial subjects received a phase-specific dose of the gamma-aminobutyric acid (GABA)-ergic general anesthetic propofol (Mhuircheartaigh, Warnaby, Rogers, Jbabdi, & Tracey, 2013). In the first phase, baseline wakeful activity was recorded for 10 min. Subjects were asked to remain still with eyes closed. In the second phase, propofol was gradually infused to a maximum effect site concentration (ESC) of 4 *μ*g/mL, which is well above the minimal dose previously found to be effective in inducing loss of consciousness in subjects and is sufficient for inducing sleep-like slow waves (Forrest, Tooley, Saunders, & Prys-Roberts, 1994). In Phase 3, the maximum ESC was maintained for 10 min, leading to a stable deep anesthesia state. In Phase 4, the propofol dose was gradually decreased until subjects regained full responsiveness. The experimental overview is shown in Figure 1A; Supporting Information Section S2 contains greater detail.

The frequency of the fMRI data was one full head volume every 3 s at a 3D voxel resolution of 2 mm^3^. All brain volumes were head extracted, artifact corrected, and registered to a single 1 mm^3^, subject-specific, high-resolution, volume scan in FSL (Jenkinson, Beckmann, Behrens, Woolrich, & Smith, 2012). The resulting volumes were mapped into the MNI152 MRI standard space. To obtain a computationally tractable anatomical partition with cortical–subcortical coverage, we used the Harvard-Oxford (HO) cortical and subcortical atlases with *D* = 63 ROIs developed in Desikan et al. (2006) and made into a combined atlas in Wilsenach et al. (2022).

In this analysis, we focus on the 10-min preinduction (Phase 1) and the 10-min postinduction recordings (Phase 3), when subjects are held at the maximum dosage. The high dosage results in a condition of deep anesthesia-induced unconsciousness and nonresponsiveness to conventional rousing stimuli. Subjects were excluded based on whether they exhibited EEG signatures that have been linked with poor tolerance for high anesthesia dosages and postoperative delirium (see Supporting Information Section S3) (Fritz et al., 2016; Z. Zhang et al., 2019). After exclusion, the total number of complete recordings for subjects in both conditions was *N* = 13.

In order to study the relationship of the brain states to the brain’s structural network, we accessed a large structural dataset combining data from 169 normative adult tractographies based on multishell diffusion tensor imaging reregistered to MNI152 standard space. The tractographies were constructed using the Gibbs tracking algorithm (Reisert et al., 2011), which were then coregistered in standard space and combined as outlined in Horn and Blankenburg (2016).

### Model Training and State Selection

HMM model selection and training for both anesthesia and wakefulness was carried out as detailed in Wilsenach et al. (2022), using the variational Bayes HMM training procedure in Vidaurre et al. (2016). This procedure is shown in Figure 1B for wakefulness (and is carried out analogously in anesthesia). As the data are high-dimensional, some compression is needed for computational efficiency. First, the high-resolution voxel-wise data are reduced by obtaining the mean BOLD signal across each ROI over the recording. For each ROI, the recordings for all subjects are then concatenated. To further reduce computational and model complexity in the HMM fitting process, a PCA was carried out to find computationally efficient combinations of ROI series, with the reduced dimension selected by parallel analysis (see Supporting Information Section S4). The reduced data were then used to train an HMM. The observations of the HMM are thus not ROIs themselves but instead combinations of ROIs. As the concatenated data obstruct the subject separation, subject-specific time series are obtained from the PCA-reduced data to infer state probabilities at each time point, while respecting the separation between subject recordings.

Following HMM training, the number of initial HMM states was selected by maximum entropy. The maximum entropy method has been shown to broadly agree with other methods of model size selection such as cross-validated maximum likelihood (Wilsenach, 2022), while often resulting in larger models that allow for more complex possible dynamics (Wilsenach et al., 2022). As this selection may result in states that are not robustly expressed, HMM states were removed if they appeared in less than 20% of subjects, and TPs were restandardized so that each row sums to one (see Supporting Information Figure S3). The (fitted) probability of each HMM state occurrence at each time point is then calculated, which determines the fractional occupancy (FO) of each state. This is the expected fraction of time spent in each state over the length of a subject recording. The resulting model for each condition has a total of *K*_*wake*_ or *K*_*anaes*_ states, respectively, and *D* ROIs.

### Graph Models of Dynamic Function and Structure

HMGMs are spatiotemporal graph models derived from the respective HMMs. More formally, an HMGM is a multiplex graph with each layer representing a brain state with the same set of ROIs as nodes, having edges determined by the functional connectivity between the ROIs, and in which the interlayer edges are the probabilities of transition between brain states. This structure can be seen in Figure 1C. HMGMs were derived from the HMMs for anesthesia and wakefulness, respectively.

A simpler, single-layer structural graph model was fit to the tractography data using DSI-Studio’s structural connectome tool (Yeh, Verstynen, Wang, Fernández-Miranda, & Tseng, 2013) and the HO atlas with default parameters and median streamline length normalization, producing an undirected, weighted graph model proxy of subject structural connectivity (Yeh et al., 2018).

### Consensus Community Detection

One of the primary strengths of graph-based models is that they allow for the decomposition of the graph into functionally related modules or communities. Modularity-based community detection is a collection of methods for finding optimal groupings of nodes (brain regions) in a graph so that members of the group are preferentially connected to one another (Girvan & Newman, 2002; Newman, 2006) in a way that numerically aims to maximize the *modularity* of the partition.

In order to determine the underlying *structural* modular organization of the structural graph model, we applied a robust version of the modularity optimization method based on Lancichinetti and Fortunato (2012) using multiple parameters and initializations to determine structural communities (see Supporting Information Section S6 for further details).

### Beyond Static Communities …

Spatiotemporal communities can be interpreted as groups of brain regions that tend to coordinate not only in space but also over time. A generalized form of modularity-based community detection was used to partition the nodes (ROIs) of the HMGM into functionally related sets that coordinate activity across space and time into dynamic functional modules (node color in Figure 1C). We determine the spatiotemporal community structure of each of the HMGM models using a new modification to the modularity that combines spatial and temporal HMM properties. The modified score takes into account both functional connectivity and temporal probabilities of transition between states (Mucha, Richardson, Macon, Porter, & Onnela, 2010; Sen, Chu, & Parhi, 2019). Using this score, communities can be interpreted as representing functional modules that can reorganize across time in order to react to external and internal stimuli. In all cases, the modularity is optimized using the generalized Louvain algorithm (Mucha et al., 2010), with the same robust aggregation over multiple parameters as in Lancichinetti and Fortunato (2012); see Supporting Information Section S6 for more detail on these methods and justification of the range of parameters. Layers with similar community composition are also clustered hierarchically; in Figure 1C, members of the same cluster are given the same color.

### Sink States, Switching and Information Rates

HMMs are probabilistic state space models in which state transitions occur probabilistically depending only on the previous state in the sequence. In a sufficiently regular Markov model, the stationary probability of a state is the long-term probability of occupying this state over a sufficiently long time (see Supporting Information Section S7). Here, we use the stationary probability to define a centrality measure over states. In analogy to PageRank centrality, we term the stationary probability of each state its *sink centrality*. As our interest lies in those states that cumulatively account for the majority of the expected time spent, in each condition, to simplify the analysis, special focus was then put on “strong” sink states that disproportionately account for activity under each condition. Here, we use as cutoff for a sink state to be considered “strong” that it has a sink centrality of at least 0.05. Other threshold choices are possible; the threshold is chosen purely to summarize the model behavior, not for training the model and is explored further in the Results section.

**Figure 2.**
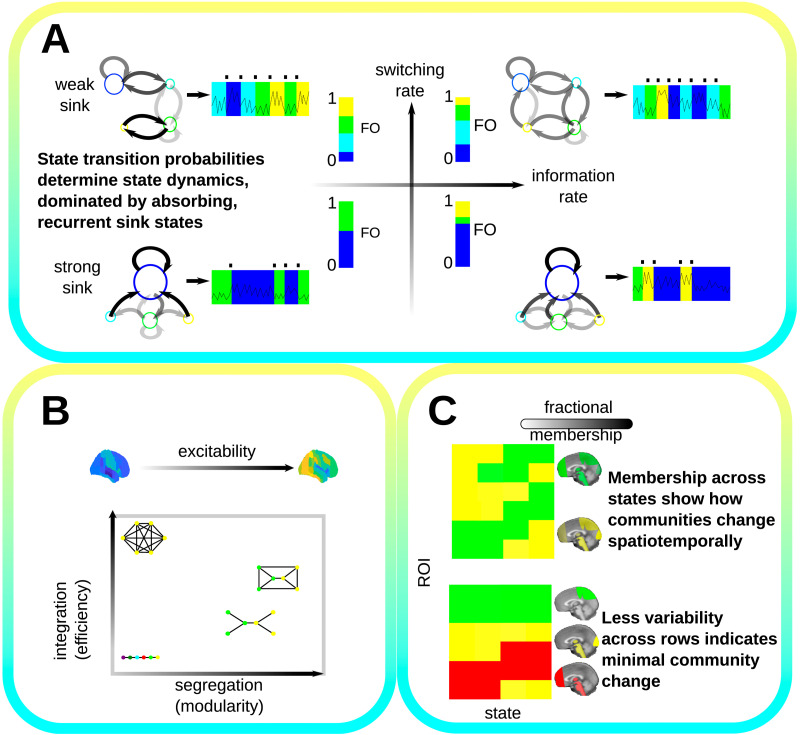
This figure shows our analysis framework (A) Four state models are shown to contrast their information theoretic properties along two axes, switching rate increasing from top to bottom and information (entropy) rate increasing from left to right. Larger nodes correspond to sinks. FO is shown as a bar plot with the length of each colored bar corresponding to the time in that state. (B) Functional variation is important to understanding conscious processing. Mean FA (excitability) may vary from low to high. The integration and segregation of functional connectivity also informs the complexity of state information processing. We measure these using efficiency (global information sharing) and modularity (capacity for independent processing), which are interdependent; for instance, the maximally integrated connectome is also minimally modular (top left), while other configurations exist somewhere in this space. (C) Spatiotemporal community analysis allows for states to be partitioned into functionally related modules that share members across temporally related states. These communities may be visualized by their fractional membership, the expected proportion of time a region spends in each community, here shown by color opacity from light (low membership) to dark (high membership).

Figure 2 provides a sketch of how HMM and HMGM analysis can provide important axes along which to examine brain state activity by contrasting model behaviors. The switching rate, Figure 2A, is a well established measure of the temporal complexity of brain state models (Stevner et al., 2019; Vidaurre et al., 2016). It is the frequency at which state change occurs in the state trajectory that best fits the data (see Supporting Information Section S8) (Brin & Page, 1998). Figure 2A shows four different four-state Markov models, where node size is proportional to the sink centrality. Edges are shaded from high (dark) to low (light) probability. A plausible state sequence is generated from each model from which the state switching events are determined and the switching rate (in hertz) is calculated. The FO is also shown, which is based on the expected time spent in each state, which is inferred directly from the model (see Supporting Information Section S1). The FO can be viewed as the distribution describing the probability of observing a subject in a given state during an entire recording.

The information (or entropy) rate measures the expected information gained by sampling from a state space model over a sufficiently long state-time (Cover & Thomas, 2006). It provides an estimate of the temporal information capacity or complexity of the underlying process (see Supporting Information Section S8), differentiating it from the switching rate, which does not account for the diversity of probabilistically realizable states. As a result, a model can have high switching rate while having relatively low complexity (Figure 2A). Brain state diversity was also measured directly using the entropy of the FO in order to consider subject-specific (rather than model or group-level) state diversity.

### Comparing States by Functional and Community Activity

HMM state functional connectivity and activity provide important modes to compare conditions of consciousness; brain activity may range from excitable (high absolute mean FA) to quiescent. We use paired distance correlation between states to compare them across both functional mean activity and functional connectivity (Figure 2B). The states themselves encode complex connectivity relationships; these high dimensional relationships are often nonlinear and in contrast to Pearson correlation, distance correlation takes into account such nonlinear dependencies (Geerligs, Cam-CAN, & Henson, 2016). Figure 2B shows a range from dark blue (quiescent) to yellow (excitable) brain states. In addition, we apply the signed distance correlation from Pardo-Diaz et al. (2021) (with the sign being the sign of the standard Pearson correlation) to provide additional information on state dependencies.

High modularity and high efficiency are both necessary properties for the brain to perform complex tasks when integration of information from multiple sources or brain regions are involved (Capouskova, Zamora-López, Kringelbach, & Deco, 2023). The competing requirements for both appropriate segregation and integration of information processing need to be balanced in order to support both homeostasis and appropriate responsiveness to stimuli (Deco, Tononi, Boly, & Kringelbach, 2015; Jang, Mashour, Hudetz, & Huang, 2024; R. Wang et al., 2021). Modularity is a proxy for segregation, with high modularity indicating a high capacity of the state to independently process multiple input streams, while high efficiency allows for fast simultaneous information sharing and integration between brain areas (Meunier, Lambiotte, & Bullmore, 2010; Sarasso et al., 2021; Zamora-López, Zhou, & Kurths, 2010). The bottom of Figure 2B shows how these two factors form a combined integration-segregation space within which functional reconfiguration occurs from state to state to meet ever changing informational processing demands on the brain.

The overall level of information integration within each state was assessed using the global efficiency of the state functional connectivity; the global efficiency measures how easily information can be propagated within a graph and is an established measure of functional integration in resting-state fMRI (Rubinov & Sporns, 2010). Efficiency calculations were normalized against efficiency values for graphs with randomly permuted edge weights in order to control for overall graph connectivity and edge strength differences between states (see Supporting Information Section S9). Modules (indicated by node colors in Figure 2B) here correspond to communities that were estimated using modularity maximization (Girvan & Newman, 2002).

Figure 2C sketches one of the primary outputs of our spatiotemporal community analysis. The fractional membership is the long run proportion of time that each brain region is expected to spend in a given community (see Supporting Information Section S10 for further details). To characterize these dynamic communities, we organize states and regions by performing hierarchical clustering using Ward’s algorithm (Ward, 1963). This process is also used to uncover common spatiotemporal patterns of functional organization across states and ROIs.

### Comparing Functional With Structural Connectivity

Patterns of functional organization are dependent upon an underlying structural connectome (Barttfeld et al., 2015; Demertzi et al., 2019; Luppi et al., 2023; Tan et al., 2019). In order to test whether deep anesthesia or wakeful state functional connectivity is more related to structural connectivity, we compared the degree and level of integration of each state across conditions to the structural connectome using distance correlation. We also used the adjusted mutual information (AMI) to quantify the similarity in community composition between structural and dynamic state models. The AMI is a measure of agreement between two partitions of brain regions that is more robust to differences in partition size than other measures such as the adjusted Rand index (Romano, Vinh, Bailey, & Verspoor, 2016) (see Supporting Information Sections S11 and S12). In order to closely compare structure and functional relationships at the level of individual brain regions, and in line with our use of global efficiency as a measure of integration we used the local efficiency first proposed in Rubinov and Sporns (2010). We employed the robust variant of the local efficiency proposed in Y. Wang, Ghumare, Vandenberghe, and Dupont (2017).

## RESULTS

### Selection of Models

We trained and analyzed two dynamic models using data from each condition. The number of states for both wakeful and deep anesthesia models was selected based on the maximum entropy of state occurrence (FO). This resulted in two different models that maximize the entropy, with 33 states for wakefulness and 19 for anesthesia, respectively (see Supporting Information Figure S2). We excluded sporadic states that featured in less than 20% of subject trajectories; this low threshold was chosen to allow for some subject-specific expression; see Supporting Information Figure S3 for the percentage of subjects visited in each state. The resulting models had *K*_*wake*_ = 32 and *K*_*anaes*_ = 18 states. For validation purposes, we also trained one combined model using both anesthesia and wakeful data concatenated to produce a single model with the same number of initial states as wakefulness, *K*_*comb*_ = 33, even after applying the same state exclusion criterion.

### Brain State Transition Graphs

**Figure 3.**
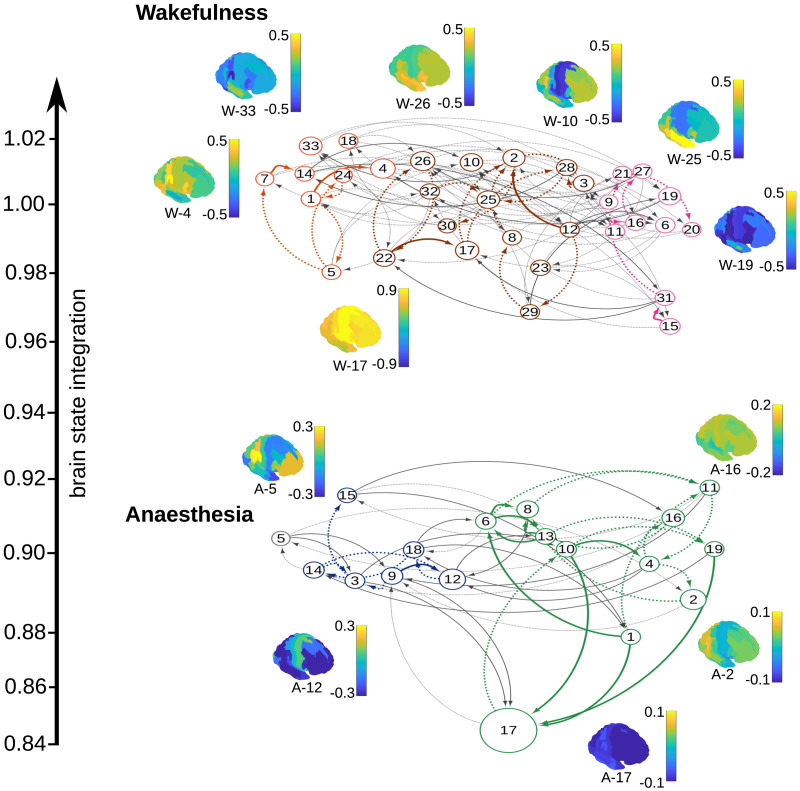
In the HMGM, states are related by the probability of transition between them. This figure shows two graphs representing the respective state dynamics of subjects when awake or anesthetized. Each node is a state the size of which is proportional to the state sink centrality. Edges represent that there is a nonnegligible TP from one state to another. For visualization purposes, only the two strongest edges from or to a node are shown with weaker probability edges (TP < 0.05) denoted by dotted lines. The level of brain state integration (normalized global efficiency) is used to order the states on the *y*-axis, showing that anesthesia states are significantly less integrated than wakeful states (*p* ≤ 0.001, Mann–Whitney *U* test). States are organized horizontally into communities with colored edges that represent connections between states that share similar functional connectivity and dynamics. Here, we show brain maps for all states that account for 0.05 or more of the stationary distribution. We show brain maps of mean FA for all sink states (and associated color bar), as well as State A-5, which is a transient outlier that does not belong to any cluster.

Figure 3 shows the TPs between states as the edges in a directed graph of states (nodes) for both anesthesia and wakefulness; wakefulness states are prefixed W-, while anesthesia states are prefixed A-. In this figure, states are organized vertically by the level of brain state integration (as measured by normalized global efficiency) and the size of states is proportional to their sink centrality. The two graph models show clear separation along the (brain) state integration axis (*p* ≤ 0.001 for the null hypothesis of global efficiency of the two models coming from the same distribution, Mann–Whitney *U* test, two-sided). The least integrated anesthesia state is A-17; this is also the strongest sink state (see Supporting Information Figure S4 for the distribution of sink centralities relative to the strong sink threshold of 0.05), with A-17 being the most dominant at 0.38 (near to 40% of expected time in any state).

Figure 3 shows that most sink states have many high-probability in-edges. In particular, A-17 is reachable with relatively high probability from states within its own cluster, and is unlikely to be transitioned to from external states, except A-3 and A-9, which both act as bridges from the other cluster. Similarly, transitions to A-12, the second strongest sink state, generally occur from within its own cluster, but there are a few states in the A-17 dominated cluster that preferentially transition to A-12 acting as bridges between clusters. State A-5 has no strong connection to either cluster and has the lowest sink centrality of any state.

Wakeful dynamics are more diverse with no dominant sink state and three separate state clusters. Mean activity maps for sink states also show larger ranges (higher excitability) than in anesthesia across all states (*p* = 0.017 ≤ 0.05 for the null hypothesis of activity being equal, measured by absolute mean activity, Mann–Whitney *U* test, two sided). Overall, the simpler modular structure of the anesthesia model suggests lower complexity dynamics and indeed the anesthesia information rate of 0.940 is less than the wakeful information rate of 1.82. Supporting Information Figure S5 shows that when varying the number of states in the model a difference between the two models persists, indicating that the difference in complexity is not simply due to a difference in the number of states but is due to the emergence of dominant states in anesthesia that affect the reachability of other brain states.

### Sink State Dominance and Anesthesia Quiescence

**Figure 4.**
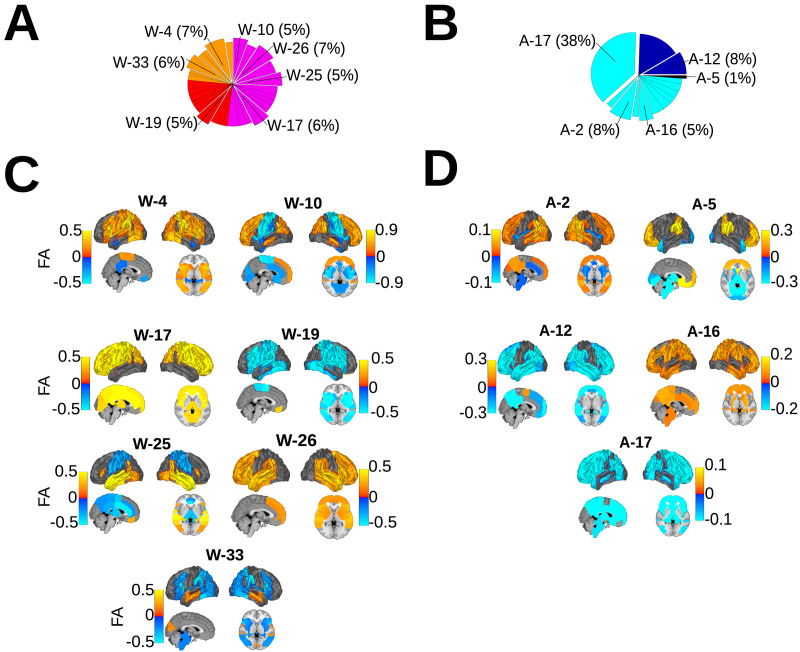
This figure summarizes the state activity of important states, highlighting the sink states as well as the outlying State A-5 and the prominent areas of surface FA for each. The stationary probability is shown for (A) wakefulness and (B) anesthesia, with wedges indicating the sink states. In (C), the top 50% mean absolute functional activity in wakefulness is shown as a surface plot with a variety of high and low activity regions. (D) This panel shows activity in the anesthesia sink states, with much lower absolute mean functional activity across these states, indicative of lower absolute mean activity in anesthesia as a whole (*p* ≤ 0.001, Mann–Whitney *U* test, two-sided).

Figure 4A and Figure 4B show the stationary distribution across all subjects as pie charts with wedges representing sink states with sink centrality at least 0.05. Again, we see strong sink state dominance in anesthesia and a more uniform stationary distribution in wakefulness. Figures 4C and 4D show the top 50% most extreme regional activity for each sink state in wakefulness and anesthesia, respectively. Nearly all regions shown for A-12 and A-17 have below-mean regional BOLD activity. In general, wakeful states have higher absolute mean activity across regions than in anesthesia (*p* ≤ 0.001, Mann–Whitney *U* test, two-sided). This also holds for the combined trial validation model (Supporting Information Figure S3) in which anesthesia trials were dominated by a single state (State 29) with lower than baseline mean absolute FA.

### Sink States Dominate Across Subjects in Anesthesia But Not Wakefulness

**Figure 5.**
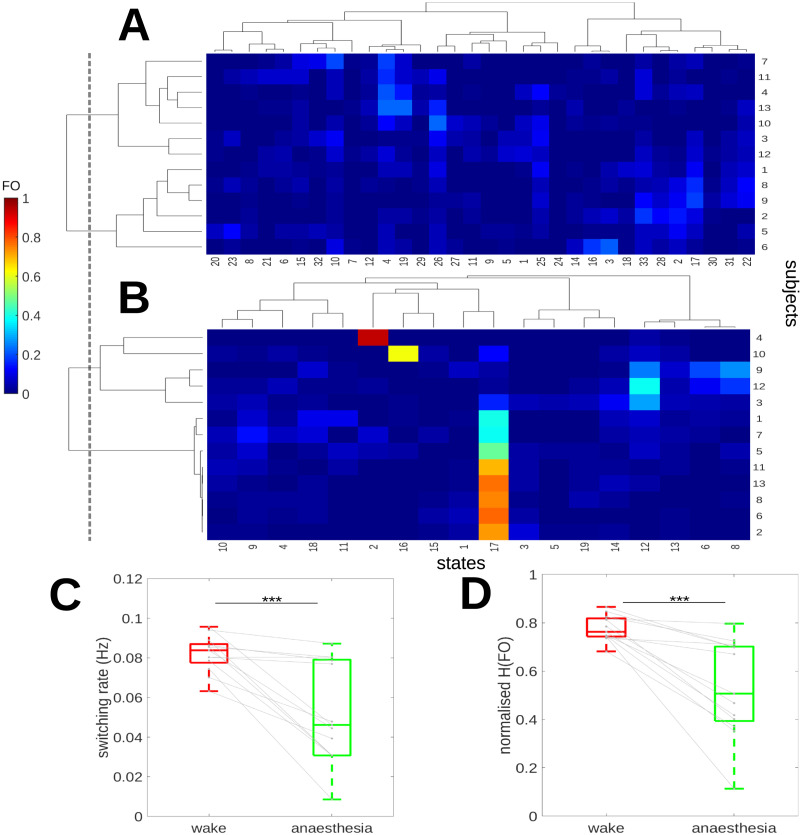
Subject-specific state dynamics are shown for both anesthesia and wakefulness. The dotted line separates both subjects into clusters according to FO correlation. (A) This panel shows the FO heat map for wakefulness as a clustergram where subjects (rows) and states (columns) have been hierarchically clustered by Pearson correlation using Ward’s algorithm. (B) In anesthesia, dynamics tend to be dominated by a subset of sink states, including Subjects 4 and 10, which show a strong preference for A-2 and A-16, respectively. (C) This panel shows a boxplot comparing wakeful and anesthetized switching rates and normalized occupancy entropy (H(FO)). The state switching rate (Hz) is more varied but significantly lower in anesthesia (*p* ≤ 0.001, Wilcoxon signed-ranks test, two-sided). The H(FO), displayed in (D), shows a similar pattern to the switching rate with highly variable but overall lower state diversity present in anesthetized versus awake subjects (*p* ≤ 0.001, Wilcoxon signed-ranks test).

Figure 5A shows the heterogeneity of state dynamics in wakefulness suggested in Figure 3. While some states do seem more prevalent among certain subject groups, for example, sink states W-4 and W-26 can be differentiated from the subject-specific patterns of sink states W-2 and W-33, no single state dominates any one grouping. By contrast, Figure 5B shows that subjects under deep anesthesia can be separated into two groups depending on sink state dominance (see Supporting Information Figure S4 for relative sink centralities across states and conditions). The majority of subjects are dominated by A-17, and the remainder have a relatively high A-12 occupancy or are occupied by subject-specific sink states (A-2 and A-16). There does not appear to be significant overlap between anesthesia and wakefulness in how subjects are grouped. It is unclear why not all subjects share the same dominant state; however, we note that the latter group is significantly younger (*p* = 0.026 < 0.05, Mann–Whitney *U* test). No significant difference in age was found between the two groups separating wakeful subjects by state FO (*p* = 0.264 > 0.05, Mann–Whitney *U* test).

The FO of the two strongest states (A-12 and A-17) have a strong negative Pearson correlation of *ρ* = −0.922 (*p* ≤ 0.01, permutation test; see Supporting Information Section S13 for details). Together, A-12 and A-17 account for ≥50% of the mean FO across all subjects in deep anesthesia, indicating that a majority of subject time is spent in one of these two states, with the overwhelming majority of time spent in A-17. This is in comparison with wakefulness in which no two states can account for more than 20% of mean FO. In the validation models using either the combined data (including both wakeful and anesthesia trials in a single model) or an alternative model with *K* = 20 states, anesthesia trials were also dominated by sink states (States 29 and 20, respectively, see Supporting Information Figures S6 and S7).

Figure 5C shows state switching rates across subjects in both conditions, with higher switching observed in wakefulness (*p* ≤ 0.001, Wilcoxon signed-ranks test, two sided). Figure 5D shows state diversity, as measured by the normalized entropy of the FO, with higher state diversity in wakeful subjects when compared with anesthesia (*p* ≤ 0.001, Wilcoxon signed-ranks test, two sided). Together, these results suggest that dynamics under deep anesthesia are not only simpler but also less dynamic, with a lower fraction of states participating in the dynamics of most subjects, as would be expected for dynamics with a lower information rate. However, there is also some evidence that subjects are more separable, for instance by state dominance, likely due to the subject-specific anesthesia depth, which varies even when subjects are held at the same ESC, and which may depend on subject-specific age and susceptibility factors (Nunes, Alonso, Castro, Amorim, & Mendonça, 2007).

### Stratification of Functional Connectivity in Anesthesia

**Figure 6.**
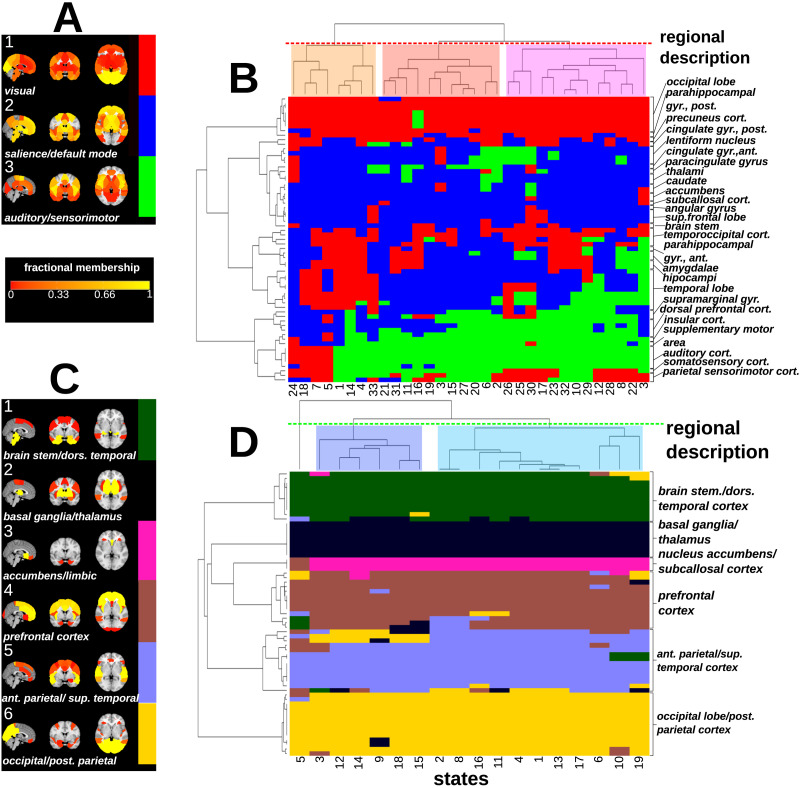
This figure summarizes the results of spatiotemporal consensus community detection across regions (rows) and states (columns). Each ROI is colored according to its community membership. ROIs and states have been hierarchically clustered in both deep anesthesia and wakefulness using the Jaccard index similarity so that states with similar community composition and regions with similar membership are co-clustered (single color patches). (A) This plot shows the regional composition of each wakeful community as a brain map where color represent the fractional membership of the region. Communities have been broadly described according to the text labels. (B) The clustergram of wakeful communities shows a heterogeneous pattern of changing community membership. (C) This panel shows brain maps for the deep anesthesia condition. Brain regions are largely static (fractional membership near to one) and have a relatively restricted anatomically organization with more total communities. (D) The anesthesia clustergram shows regional stratification with minimal reorganization.

In the wakeful condition, state diversity results in highly diverse spatiotemporal community reorganization. Hierarchical clustering of spatiotemporal community membership, combining functional and temporal relationships, reveal highly distributed dynamics, with each community including diverse and dynamically changing membership (see Figure 6A). These communities dynamically represent shifts between resting-state networks including visual, salience-default mode and sensory networks respectively. The dynamics is visible in Figure 6B, where regional switching between communities is reflected in the mosaic pattern of community associations.

The anesthesia model (see Figures 6C and 6D) reveals strikingly different results, with a greater number of less dynamic communities that are primarily restricted to closely anatomically related ROIs. There is far less change of membership between states in anesthesia in comparison with wakefulness, resulting in stratified and near static dynamic reorganization. Even though we see less switching in anesthesia dynamics, sporadic co-recruitment of regions commonly associated with the so-called resting-state networks does occur, for example, in A-16 (exhibiting default mode-like activity in Community 3).

Furthermore, the spatiotemporal hierachical clustering of communities in anesthesia suggests that subcortical areas, most notably the thalamus and neighboring regions (including the caudate), generally thought of as an information bottleneck for sensory inputs reaching the brain (Alitto & Usrey, 2003; Luppi et al., 2024), are isolated from cortical brain regions in almost all anesthesia states. This is not seen in wakefulness, in which thalamic regions share and switch membership with multiple cortical regions depending on state, supporting the dynamic role of the thalamus in conscious homeostasis.

### Sink State Similarity Across Conditions

**Figure 7.**
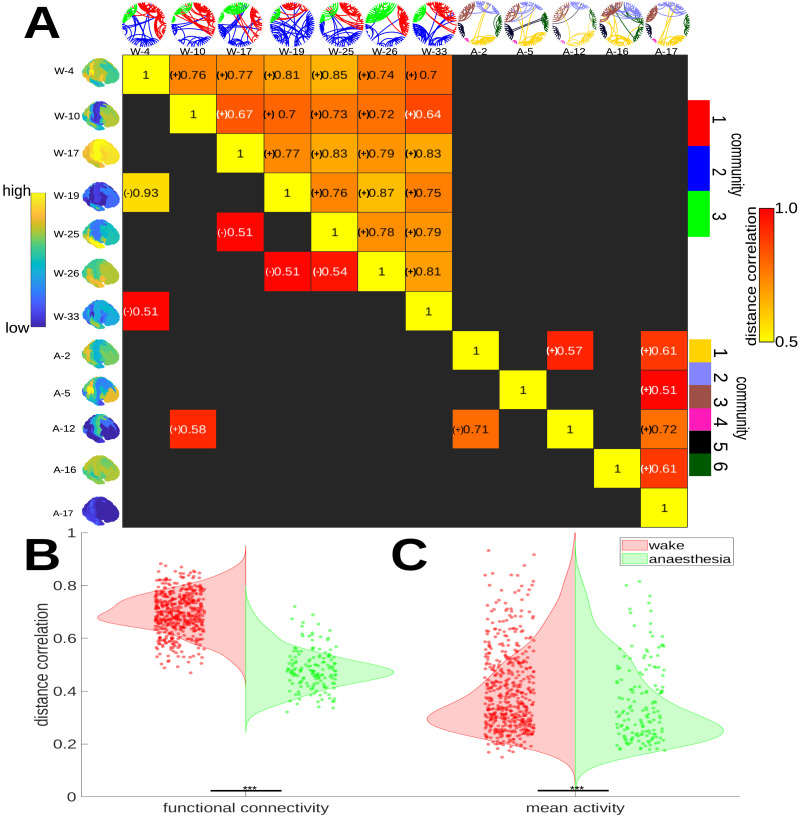
This figure illustrates the functional mean activity and connectivity across sink states in anesthesia and wakefulness. (A) This figure is a heatmap of the pairwise signed distance correlation between sink states functional activity (bottom left) and sink state connectivity (top right). Brain maps (left) are the same as those in Figure 3. Community interconnectivity (top) is shown as a circular graph, depicting the level of intercommunity connectivity in each state. (B) This violinplot shows the distance correlation in functional connectivity between pairs of states (dots) in (red) wakefulness and (green) anesthesia (Mann–Whitney *U*, *p* ≤ 0.001, two-sided). Crosses denote outlying states. (C) Signed distance correlation is higher for mean activity between wakeful states although the difference appears smaller (Mann–Whitney *U*, *p* ≤ 0.001, two-sided).

Plots of the functional connectome of each of the sink states are shown in Figure 7A (thresholded to the top 5% strongest connections by magnitude for illustrative purposes), with mean activity maps for each state shown vertically. The heatmaps represent the pairwise distance correlation (displaying only strong relationships, >0.5) between states across both conditions. Very few wakeful states share a significant relationship with any anesthesia state on the basis of either FA (bottom left) or connectivity (top right). Figure 7B shows that pairwise distance correlation of functional connectivity between wakeful states is significantly higher than in anesthesia (*p* ≤ 0.001, Mann–Whitney *U* test, two-sided). This difference in state self-similarity holds even when considering distance correlation in signed functional (Pearson) correlation between ROIs (*p* ≤ 0.001, Mann–Whitney *U* test, two-sided). There is evidence for the suggestion that most anesthesia states resemble neither each other nor are directly related to wakeful states, even when considering possible changes in the sign of functional relationships. A similar but weaker finding holds for FA (*p* ≤ 0.001, Mann–Whitney *U* test, two-sided) with mean FA distance correlation lower in anesthesia than in wakefulness; see Figure 7C. Although anesthesia states are generally superficially dissimilar, signed distance correlation does suggest that A-17 is at least somewhat positively related to all other anesthesia states; this relationship between anesthesia states makes it plausible to observe the perhaps surprising similarity in spatiotemporal community composition seen across anesthesia states.

### Structure–Function Relationships in Wakefulness and Anesthesia

**Figure 8.**
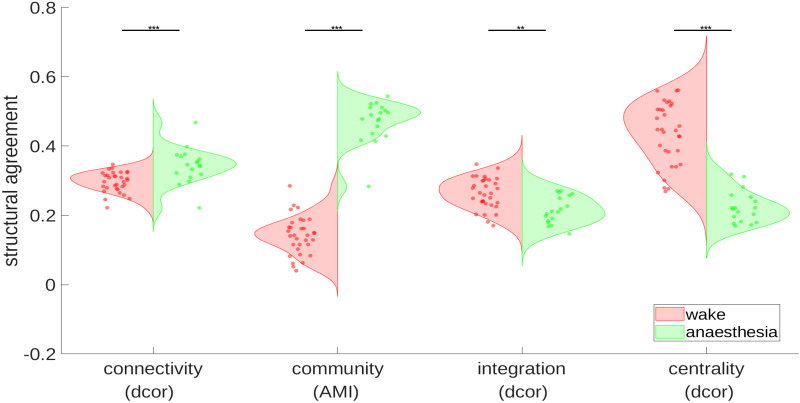
This figure shows the differences between brain state functional connectivity in wakefulness and anesthesia as they relate to structural connectivity using either distance correlation (dcor) or AMI, visualized as a violinplot with states represented as either red (wakefulness) or green (anesthesia) dots. The connectivity plot shows the relationship between brain state edge weights and structural network edge weights, with anesthesia state connectivity being more similar to structural connectivity (*p* = 0.001, Mann–Whitney *U* test, two-sided). The community violinplot shows the AMI, a measure of modular similarity, between brain state modules and structural modules suggesting that anesthesia functional modules are more similar to the normative structural modularization of the brain than more dynamic wakeful states (*p* < 0.001, permutation test). For local comparison, integration is here measured by the local efficiency compared with the structural local efficiency (*p* ≤ 0.001, Mann–Whitney *U* test, two-sided). Centrality shows the distance correlation in degree centrality between structure and state graphs (*p* ≤ 0.001, Mann–Whitney *U* test, two-sided).

Similarity analysis of the functional connectivity of each state with the underlying structural connectivity reveals significant differences in how wakeful and anesthesia states relate to or agree with (under a given similarity measure) structural connectivity. The connectivity violinplot in Figure 8 shows the distance correlation (dcor) between edge weights of a state and structural edge weights (each expressed as vectors). The community violinplot in Figure 8 shows the results of comparing structural and functional modules in both conditions, as determined by consensus multiresolution community analysis (Supporting Information Figure S8 for these modules). Here, we used the AMI, a standard measure of community agreement, to determine similarity in community organization between brain states and structural organization. Anesthesia community composition and connectivity are moderately consistent with the observed structural modularization, having an average AMI of 0.51 between states, when compared with wakefulness, having an AMI of 0.24 (*p* ≤ 0.001, permutation test); we also validated these results using the ARI (see Supporting Information Sections S11 and S12). Wakefulness was found to be more correlated to structure in terms of local efficiency (integration) and degree centrality statistics (*p* ≤ 0.001, Mann–Whitney *U*, two-sided). The connectivity and degree centrality results hold even when signed Pearson correlation is used in place of the unsigned (absolute) connectivity we use in our framework (*p* ≤ 0.001, Mann–Whitney *U*, two-sided).

### Sink State Occupancy Correlates With Age and Susceptibility

Given the overwhelmingly dominant role of the strongest sink state in determining the range of anesthesia brain state dynamics, it is important to determine which factors, if any, are most determinative of sink state dominance. Multivariate linear regression was performed to determine which factors most closely predict the time subjects spent (FO) in the most dominant sink state A-17. Increasing age was found to be the single variable most associated with positive increases in A-17 FO (*p* = 0.005 ≤ 0.01, *t* test, two-sided) when considered alone. However, time at loss of responsiveness (*T*_*LOBR*_) in combination with age was found to be the best performing predictor of A-17 FO with respect to both the BIC (see Supporting Information Table S2) and adjusted *R*^2^ criteria, with significant improvements in goodness of fit over the age-only model (*p* = 0.040 ≤ 0.05, chi-squared test). The model shows that age is a significant predictor (*p* = 0.004 ≤ 0.01, *t* test, two-sided), while *T*_*LOBR*_ is marginally negatively associated with A-17 FO (*p* = 0.079, *t* test, two-sided). Supporting Information Figure S9 shows the alternative model, which includes time conditioned on age. Surprisingly, gray matter density (see Supporting Information Section S14) did not add any significant model improvement (see Supporting Information Tables S3 to S6).

## DISCUSSION

In this paper, we contrast the dynamics of resting wakefulness and unconsciousness under deep anesthesia using data from a multiphase study of subjects undergoing propofol-induced anasthesia, focusing on analysis of fMRI BOLD activity. In the analysis, we apply a combined HMM-based dynamic state space model (Vidaurre et al., 2016), as well as a new spatiotemporal community detection approach, in order to determine the dynamic modular reorganization of brain activity. We provide results corroborating a general decrease in temporal complexity during unconsciousness seen in other investigations (Barttfeld et al., 2015; Demertzi et al., 2019; Mediano et al., 2023), and propose that this dynamic may be due to the dominance of low-complexity sink states and widespread functional disintegration. We divide these results into three primary findings that we discuss here.

First, regarding the brain state dynamics, we corroborate and expand on existing research showing that unconscious dynamics (Mediano et al., 2023; Stevner et al., 2019), and anesthesia dynamics in particular (Barttfeld et al., 2015; Demertzi et al., 2019; Hudetz, Liu, & Pillay, 2015; Liang et al., 2024), are temporally less complex both in terms of the effective diversity and reachability of states and in state switching. We suggest that the attraction of anesthesia dynamics toward a small set of sink states, with a severely reduced capacity for integration and cortical and subcortical excitability, may, in part, be responsible for this reduction in complexity. However, visits to other states may suggest moments of brief burst suppression, partial arousal in responses to external or internal perturbations (Dueck et al., 2005; Mhuircheartaigh et al., 2013; Z. Zhang et al., 2019), or even commonly reported dreamlike events (Hack et al., 2024). Sink state dominance seems much less prevalent in wakefulness with comparatively much weaker attractorlike states, perhaps owing to a need for higher levels of responsiveness.

Second, we observe fragmentation of functional modules under anesthesia into small, static subnetworks with little thalamocortical or even cortical–subcortical reorganization from state to state. In particular, thalamic community associations mostly occurred between neighboring subcortical and entorhinal regions. Notably, some interchange is observed between prefrontal, parietal, and nucleus accumbens, suggesting some sporadic default mode-like activity. This thalamocortical isolation has also been shown from static measures of functional connectivity (Mhuircheartaigh et al., 2013). Deteriorated but persistent default mode network activity has been observed in other studies of anesthesia functional connectivity, with the anterior portions being most affected (Luppi et al., 2019). In our model, anesthesia is contrasted with wakefulness in which larger communities dynamically exchange members between states to facilitate more complex task performance, required for conscious awareness and for responsiveness to the external and internal environment (Barttfeld et al., 2015). This result may be surprising as we find that anesthesia state connectivity was generally less self-similar than in wakefulness. However, this is consistent with the hypothesis that there is a persistent global core to functional connectivity in wakefulness (Onoda & Akama, 2023), likely including the thalamus as an important hub (Luppi et al., 2024; Mhuircheartaigh et al., 2013; Tasserie et al., 2022). Stratification may result in this core being partially fragmented into anatomically restricted regions during unconsciousness (Hu, Wang, Zhang, Luo, & Wang, 2024).

Third, we examine the dynamic structure–function relationship in relation to both connectvity and modulariztion. Our results support the generally observed increase in similarity between functional and structural connectivity as measured by connection weights (Barttfeld et al., 2015; Demertzi et al., 2019), as well as providing new evidence of weak similarity between dynamic modularization and structural modularization under anesthesia. In particular, we note that most anesthesia communities appear spatially constrained to specific brain areas, perhaps reflecting propagation of anesthesia signal along short-range structural connections that then fail to propagate more widely as observed in Gosselin, Bagur, and Bathellier (2024) and Mhuircheartaigh et al. (2013). Long-range structural connections have been shown to be sparser and more targeted in comparison with short-range connections (Betzel & Bassett, 2018), supporting diverse, complex functional processes in wakefulness that may be absent in unconsciousness and in severe cases of DOCs (Altmayer et al., 2024; Luppi et al., 2021, 2025). In addition to anatomical modularization, this difference between short- and long-range connectivity may explain the dissimilarity in efficiency and degree centrality between anesthesia states and underlying structural connectivity.

Finally, we indicate some implications of our study regarding patient-specific targeting of anesthesia depth. The strong attractorlike dynamics toward low-complexity, low-excitability anesthesia states that we observe reflect the imperturbability of subjects from anesthesia. The association of specific brain states with levels of arousal under anesthesia has previously been reported (Barttfeld et al., 2015). We demonstrate subject-specific sink state activity with changes in the degree of sink state dominance and the specific sink state active, defining possible differences in the steady state for anesthesia homeostasis, which may indicate differing capacities for information integration and arousal. We find a tentative link between sink state dominance and anesthetic susceptibility, specifically the age of the subject, and, to a lesser extent, time at loss of responsiveness. This may relate to previous evidence of age-related state activity in surgical EEG (Li et al., 2020). Furthermore, these results suggest that sink state dominance may share similar features with common monitors of anesthesia depth that are also known to be modulated by age and susceptibility-specific factors (Cortínez et al., 2008; Obert et al., 2021; Warnaby, Sleigh, Hight, Jbabdi, & Tracey, 2017). More tentatively, the persistence of sink state-like dynamics may be a possible future marker for recovery from DOCs.

With regard to limitations, our study was limited in its structure–function comparisons by the availability of patient-specific structural data that would enable us to make subject-specific comparisons. Finding a parcellation that was computationally tractable and relevant in a functional context was prioritized partially due to the relatively small cohort size for the ultraslow anesthethesia study. Due to this choice, some cortical areas lacked comparable left and right regions, preventing us from observing possible lateralization of activity in some cases. Sample size and small age range may also explain the apparent weak association between gray matter volume and age (Terribilli et al., 2011), and limit the generalizability of the result to older ages who see the most significant detrimental affects of over anesthesia (Sevenius Nilsen et al., 2019; Wu et al., 2019). In this regard, further experimental studies would be of interest.

The focus on brain state has allowed for the development of new ways of analyzing the complexity of the brain in action (Barnett & Seth, 2023; Barttfeld et al., 2015; Mediano et al., 2022). We believe that new complexity measures should put special focus on combining both spatial and temporal complexity while taking into account the brain’s dynamic capacity for integrating the interdependent streams of information that support complex behavior. Our HMGM approach provides a novel graphical solution to address the problem by decomposing the entire neural system into spatiotemporal subnetworks that capture the reorganization necessary to sustain complex activity.

## ACKNOWLEDGMENTS

The authors are particularly grateful to Marco Fabus for his assistance in identifying periods of burst suppression from subject EEG and Minh Tran for his helpful advice in model design and testing. The authors are grateful to the following funding bodies: G. R. is funded in part by the UKRI EPSRC Grants EP/T018445/1, EP/R018472/1, EP/X002195/1, and EP/Y028872/1, J. W. is funded by the UKRI BBSRC Grant BB/Y003020/1 and K. W. acknowledges funding from the UKRI MRC Grant MR/R006423/1. For the purpose of Open Access, the authors note that a CC BY public copyright license applies to any author accepted manuscript version arising from this submission.

## SUPPORTING INFORMATION

Supporting information including supporting figures and method details are included in the Supporting Information document.

## AUTHOR CONTRIBUTIONS

James Barnard Wilsenach: Formal analysis; Investigation; Methodology; Project administration; Validation; Visualization; Writing – original draft; Writing – review & editing. Charlotte M. Deane: Investigation; Methodology; Project administration; Resources; Software; Supervision; Writing – original draft. Gesine Reinert: Funding acquisition; Methodology; Project administration; Resources; Software; Supervision; Writing – original draft; Writing – review & editing. Katie Warnaby: Conceptualization; Data curation; Funding acquisition; Investigation; Methodology; Project administration; Resources; Software; Supervision; Writing – original draft.

## FUNDING INFORMATION

James Barnard Wilsenach, Biotechnology and Biological Sciences Research Council (https://dx.doi.org/10.13039/501100000268), Award ID: BB/Y003020/1. Gesine Reinert, Engineering and Physical Sciences Research Council (https://dx.doi.org/10.13039/501100000266), Award ID: EP/T018445/1. Gesine Reinert, Engineering and Physical Sciences Research Council (https://dx.doi.org/10.13039/501100000266), Award ID: EP/R018472/1. Gesine Reinert, Engineering and Physical Sciences Research Council (https://dx.doi.org/10.13039/501100000266), Award ID: EP/X002195/1. Gesine Reinert, Engineering and Physical Sciences Research Council (https://dx.doi.org/10.13039/501100000266), Award ID: EP/Y028872/1. Katie Warnaby, Medical Research Council (https://dx.doi.org/10.13039/501100000265), Award ID: MR/R006423/1.

## Supplementary Material



## TECHNICAL TERMS

HMM: Hidden Markov model, a probabilistic model of (brain) state that defines a model for observed data and the transitions between (brain) states.
HMGM: Hidden Markov graph model, an augmented HMM with the structure of a multiplex graph.
Functional module: A set of two or more brain regions which coordinate to perform a specific cognitive function (these modules may overlap in time and/or space).
Propofol: An intravenous anesthetic commonly used in general anesthesia for surgical purposes.
BOLD: Blood oxygen level-dependent (signal), a measure of functional activity under fMRI or other imaging modalities.
ROI: Region of interest, a defined region of the brain for investigation in the study.
Community: A collection of nodes in a network that are functionally coordinated across space and/or time.
LOBR: Loss of behavioral responsiveness, the individualized point at which subjects stop responding to experimentally relevant stimuli.
ESC: Effect site concentration, the concentration of a drug where it is biologically active.
Tractography: The use of imaging data to model the connectivity of the axon bundles that physically connect brain regions.
FO: Fractional occupancy, the fraction of time that a subject is expected to spend in each state, given their functional activity, over a specific recording.
Multiplex: A network comprised of copies of the same set of nodes (layers) with edges between them.
Modularity: A measure of the degree of coordination between members of communities when compared with the expected degree of coordination between those members.
Parcellation: A 3D brain model that defines certain brain voxels as belonging to distinct brain regions.
